# Supplementary material for: Genome-wide association studies and fine-mapping identify genomic loci for n-3 and n-6 polyunsaturated fatty acids in Hispanic American and African American cohorts
Source: Commun Biol. 2023 Aug 16;6:852. doi: 10.1038/s42003-023-05219-w (PMC10432561; doi:10.1038/s42003-023-05219-w)
Supplement: Supplementary file 3 — Description of Additional Supplementary Files [file 42003_2023_5219_MOESM3_ESM.docx]

**Description of Additional Supplementary Files**

**File name:** Supplementary Data 1

**Description:** Examination of variants identified in prior CHARGE European American GWAS of PUFAs

**File name:** Supplementary Data 2

**Description:** Credible sets of putative causal variants for each of the PUFAs in Hispanic Americans

**File name:** Supplementary Data 3

**Description:** Credible sets of putative causal variants for eachof the PUFAs in African Americans

**File name:** Supplementary Data 4

**Description:** Chromatin Contact Analysis for Understanding the Chromatin Interaction on Chromosome 11

**File name:** Supplementary Data 5

**Description:** Cross-ancestry Replication for lead variants from credible sets of putative causal variants in Hispanic Americans

**File name:** Supplementary Data 6

**Description:** Cross-ancestry Replication for lead variants from credible sets of putative causal variants in African Americans

**File name:** Supplementary Data 7

**Description:** Source data for Figure 3b

**File name:** Supplementary Data 8

**Description:** Association with lipid traits for lead variants from credible sets of putative causal variants in Hispanic Americans

**File name:** Supplementary Data 9

**Description:** Association with lipid traits for lead variants from credible sets of putative causal variants in African Americans

**File name:** Supplementary Data 10

**Description:** Colocalization analysis using MESA multi-ancestry eQTL in Hispanic Americans

**File name:** Supplementary Data 11

**Description:** Colocalization analysis using GTEx multi-ancestry eQTL in Hispanic Americans

**File name:** Supplementary Data 12

**Description:** PrediXcan results using MESA expression prediction models in Hispanic Americans

**File name:** Supplementary Data 13

**Description:** PrediXcan results using GTEx expression prediction models in Hispanic Americans

**File name:** Supplementary Data 14

**Description:** Gene Co-expression analyses using GTEx whole blood expression to examine candidate genes from Integrative analyses

**File name:** Supplementary Data 15

**Description:** Gene set enrichment analysis for genes implicated by colocalization and PrediXcan analysis
