# Supplementary material for: Genome-wide association studies and fine-mapping identify genomic loci for n-3 and n-6 polyunsaturated fatty acids in Hispanic American and African American cohorts
Source: Commun Biol. 2023 Aug 16;6:852. doi: 10.1038/s42003-023-05219-w (PMC10432561; doi:10.1038/s42003-023-05219-w)
Supplement: Supplementary file 2 — Supplementary Information [file 42003_2023_5219_MOESM2_ESM.pdf]

Supplementary Figure 1. Manhattan plot of meta-analysis of GWAS for PUFAs in the Hispanic Americans.
The red line shows the genome-wide significance threshold of ($-\log_{10}(5 \times 10^{-8})$).

AA

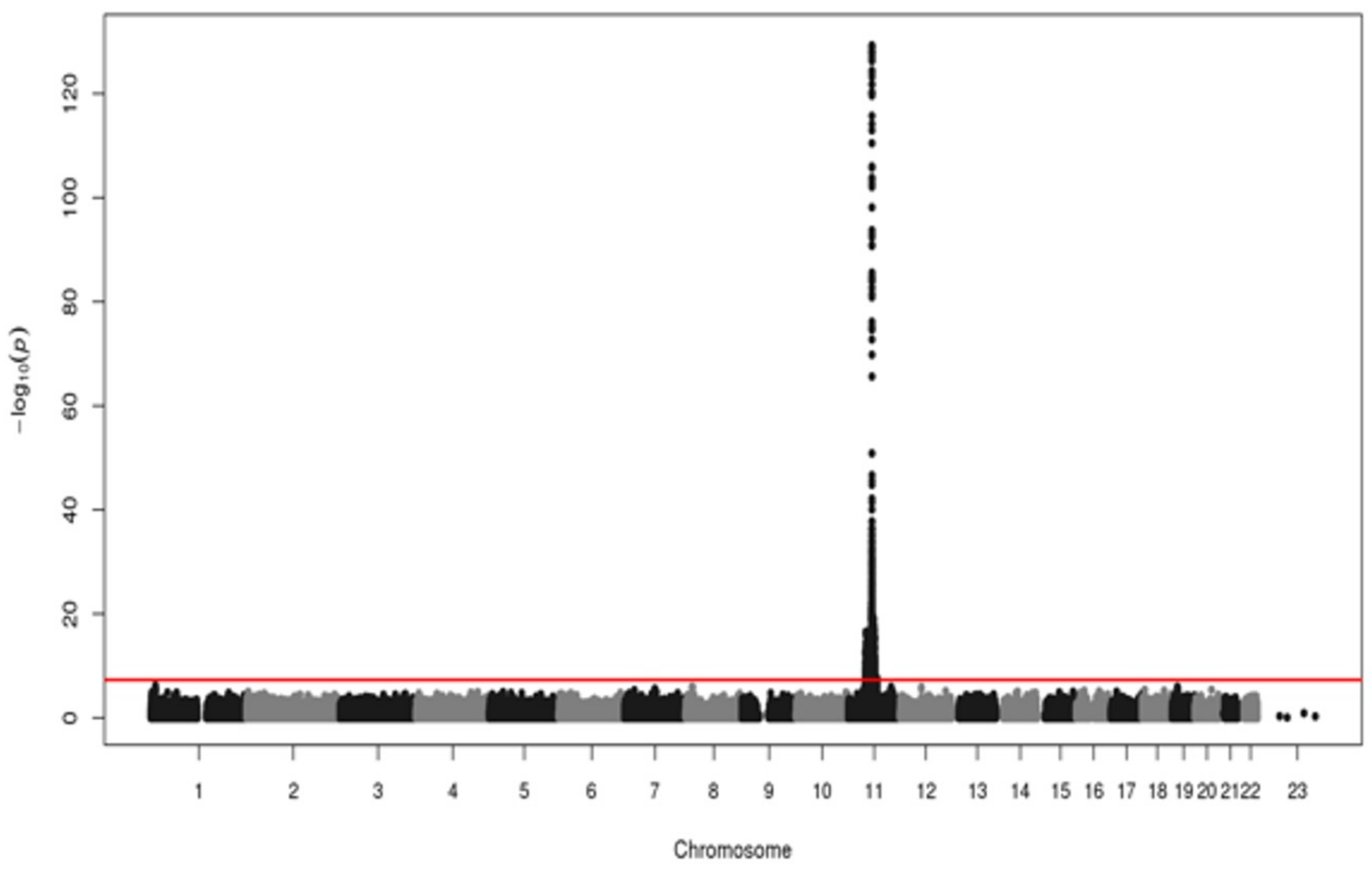

ALA

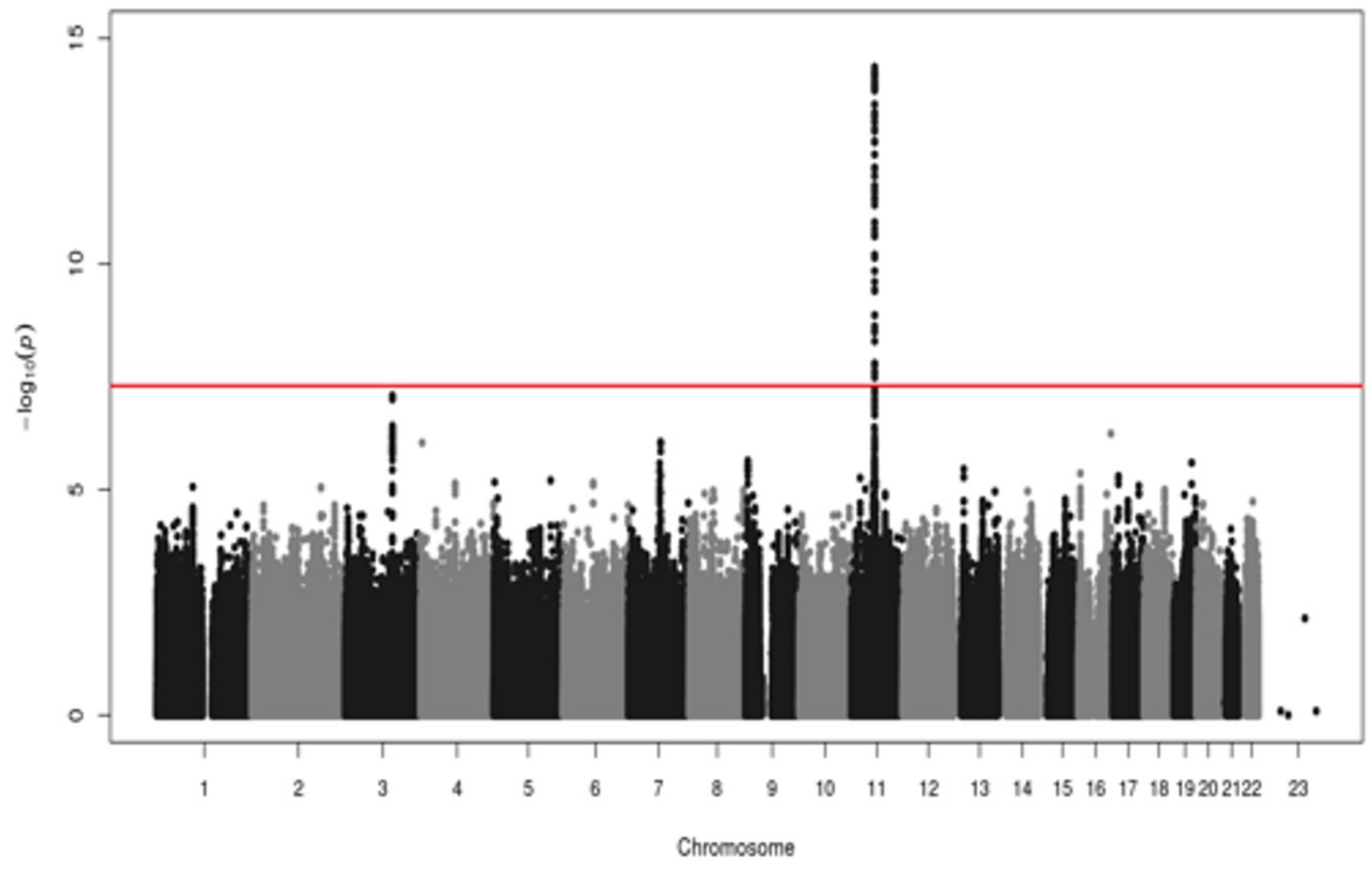

DGLA

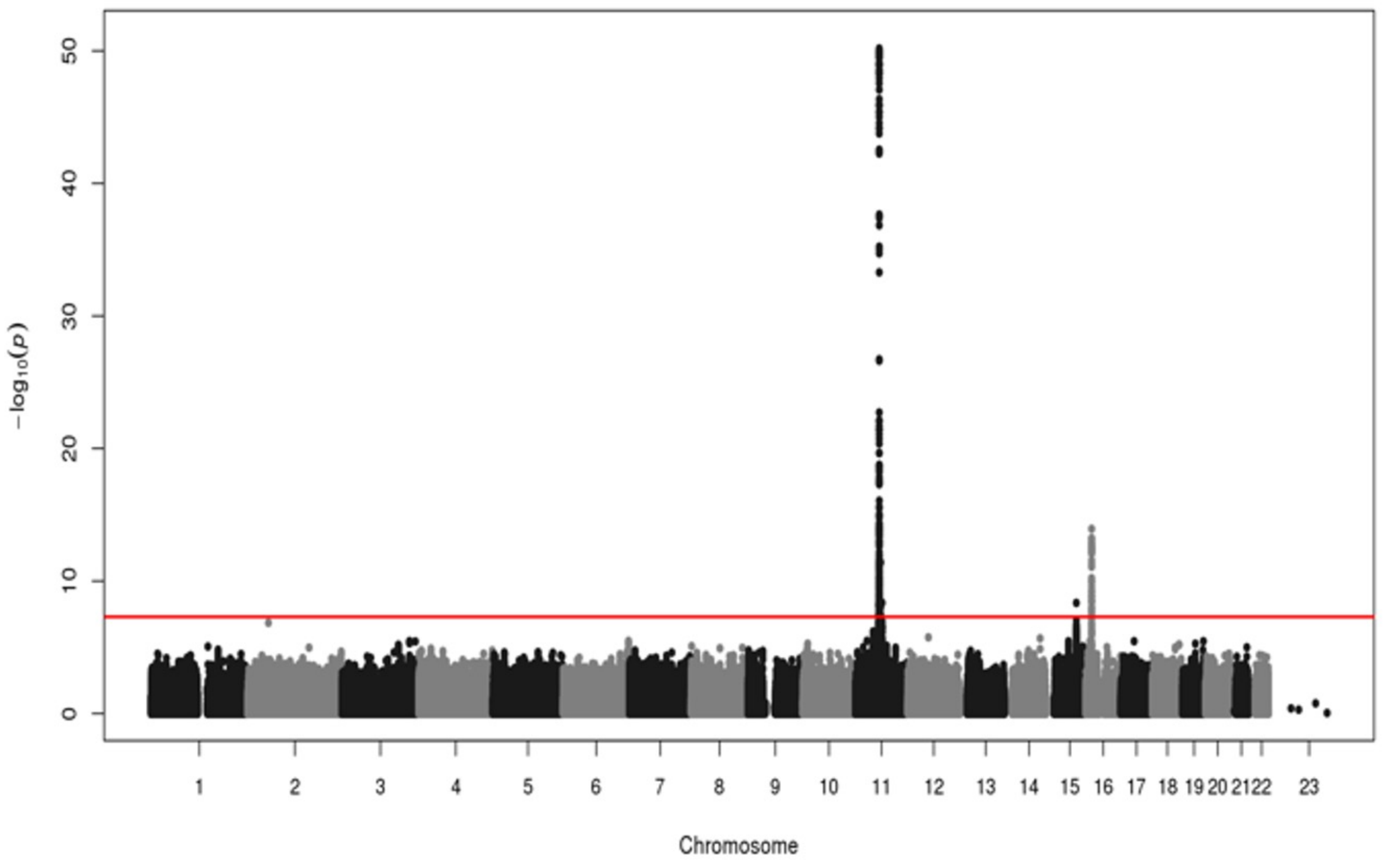

DPA

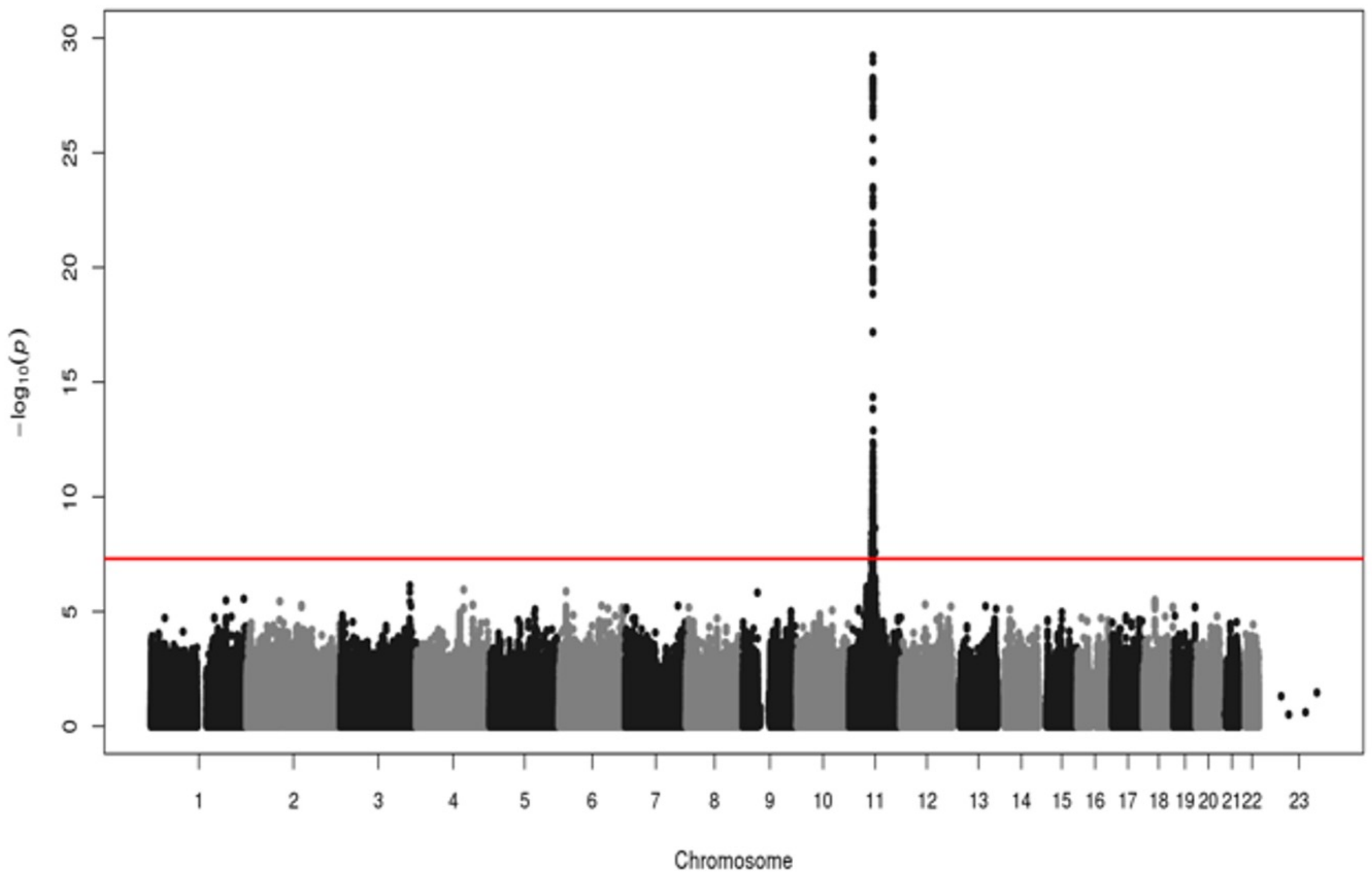

EPA

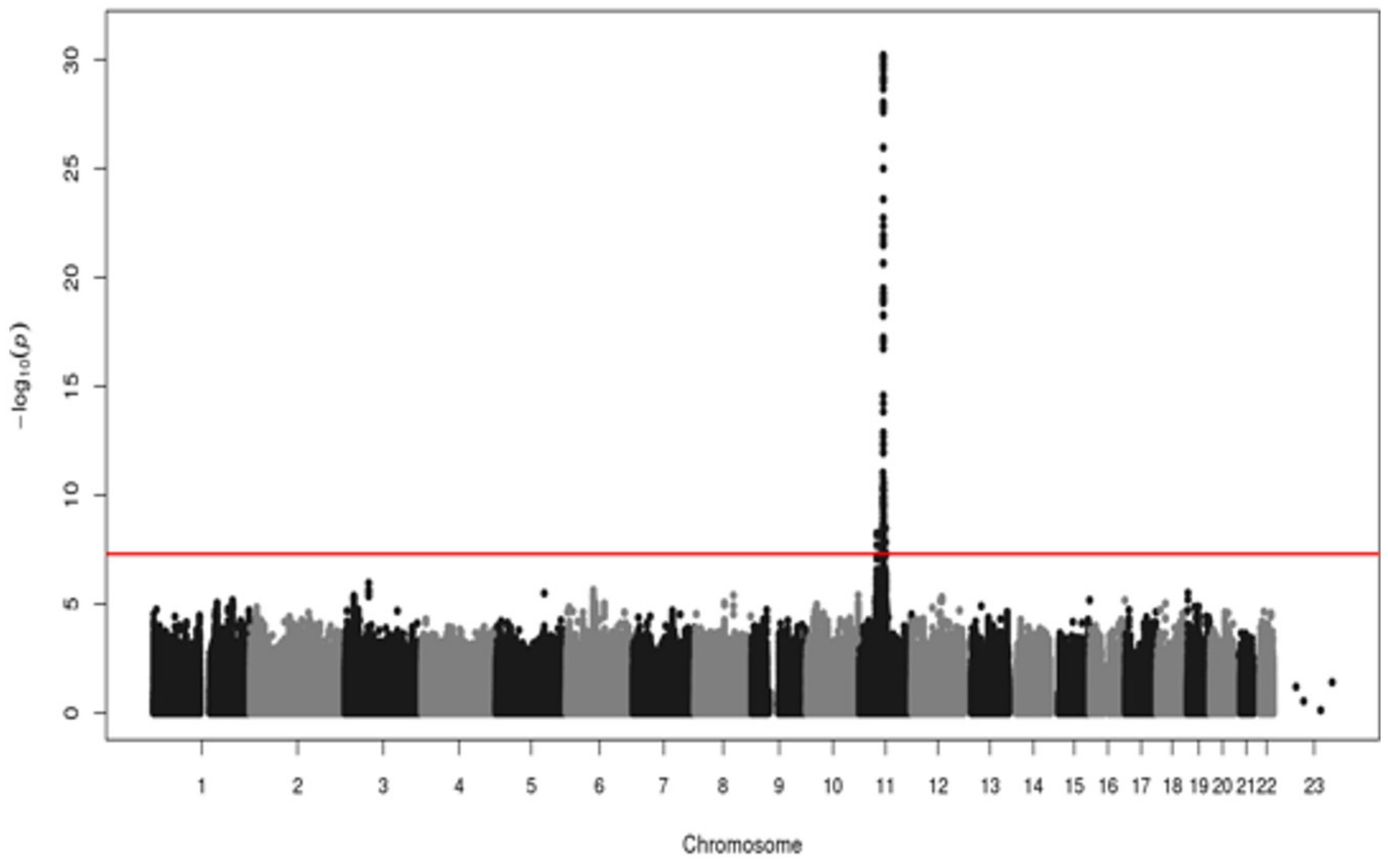

GLA

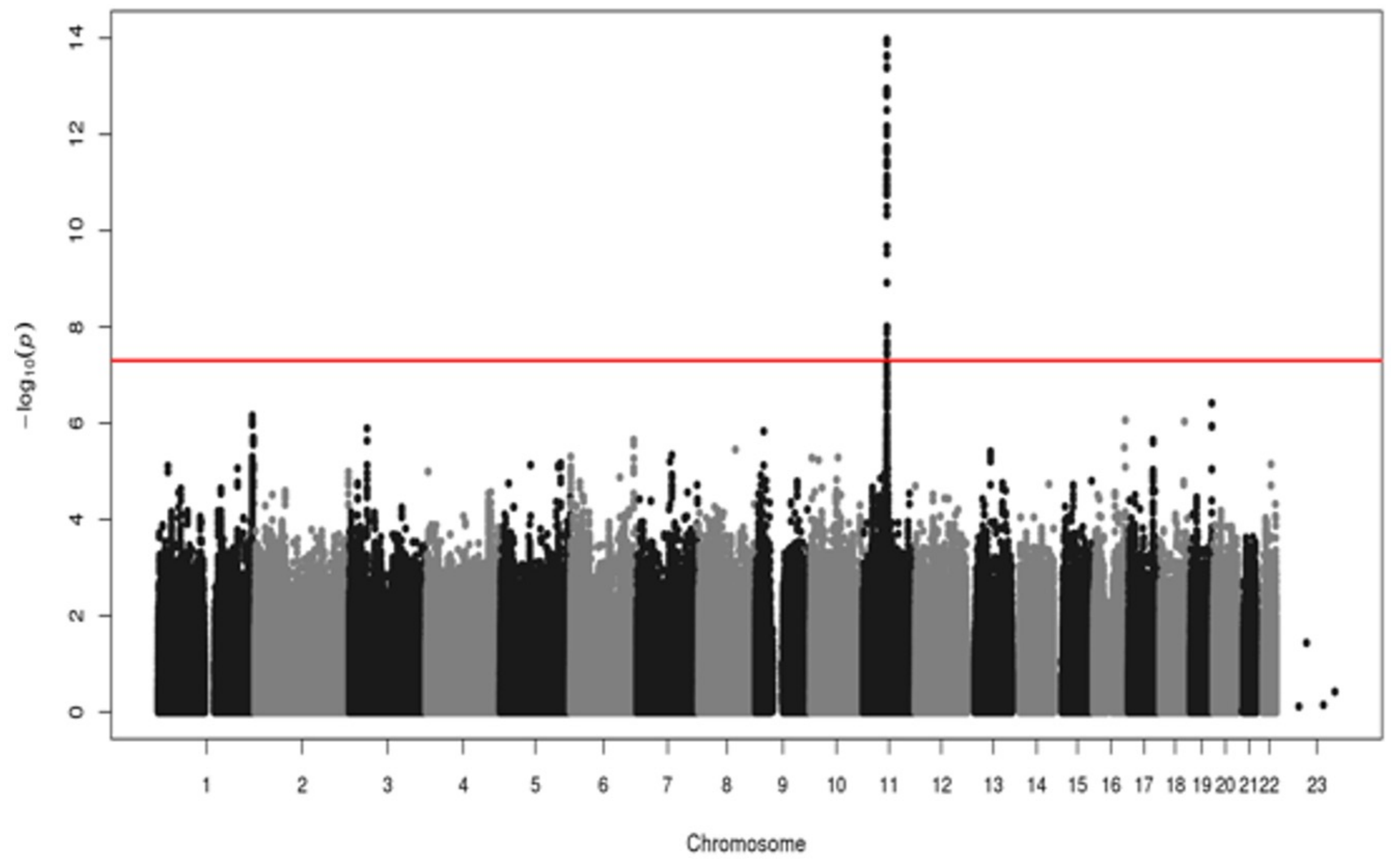

LA

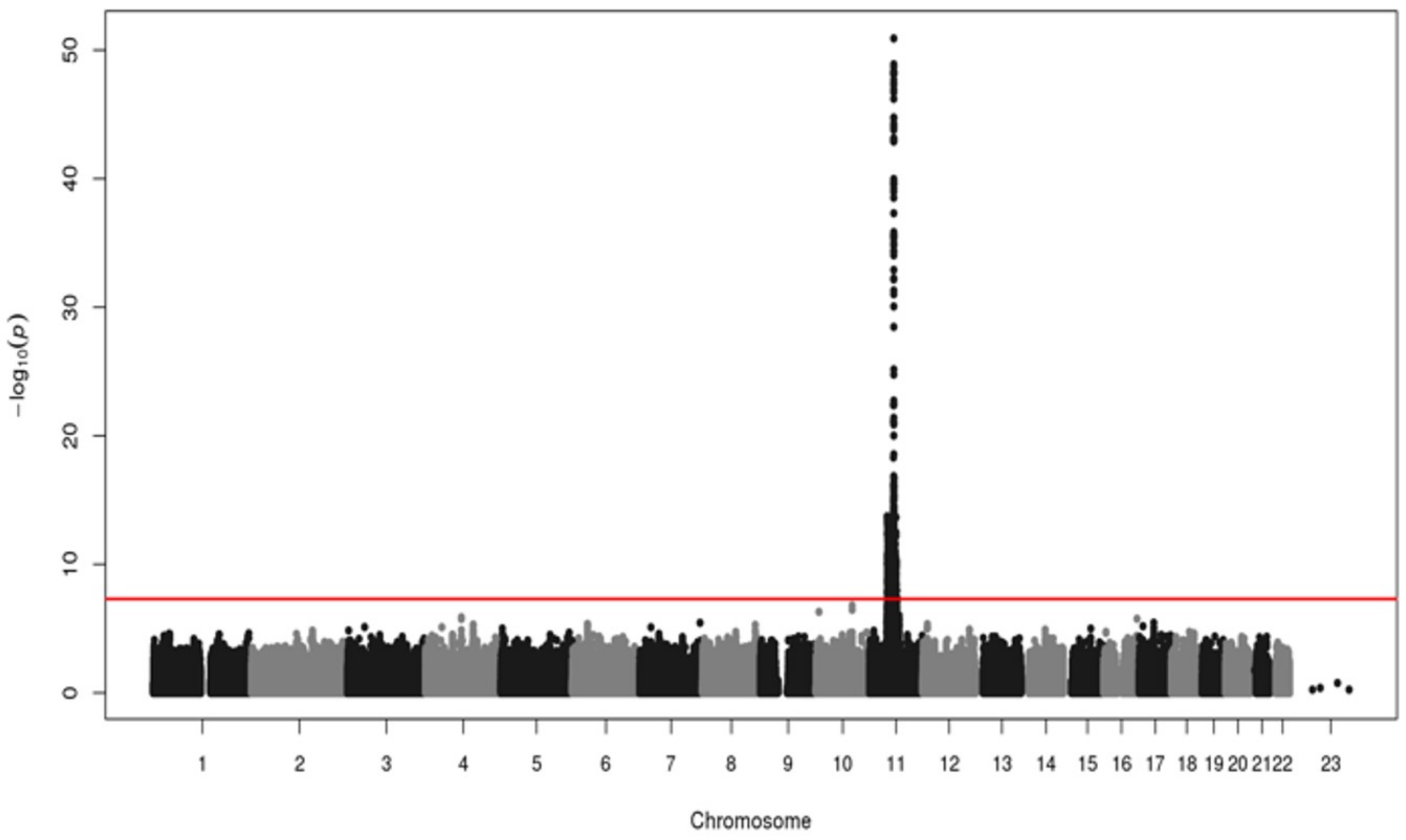

Supplementary Figure 2. Local association plot of most significant for each PUFAs on each chromosome in the Hispanic Americans.

Reference panel used to color LD is 1000 Genomes Ad Mixed American (AMR) and the color scheme is red for strong linkage disequilibrium (LD; $r^2 \geq 0.8$) and blue color for lower LD.

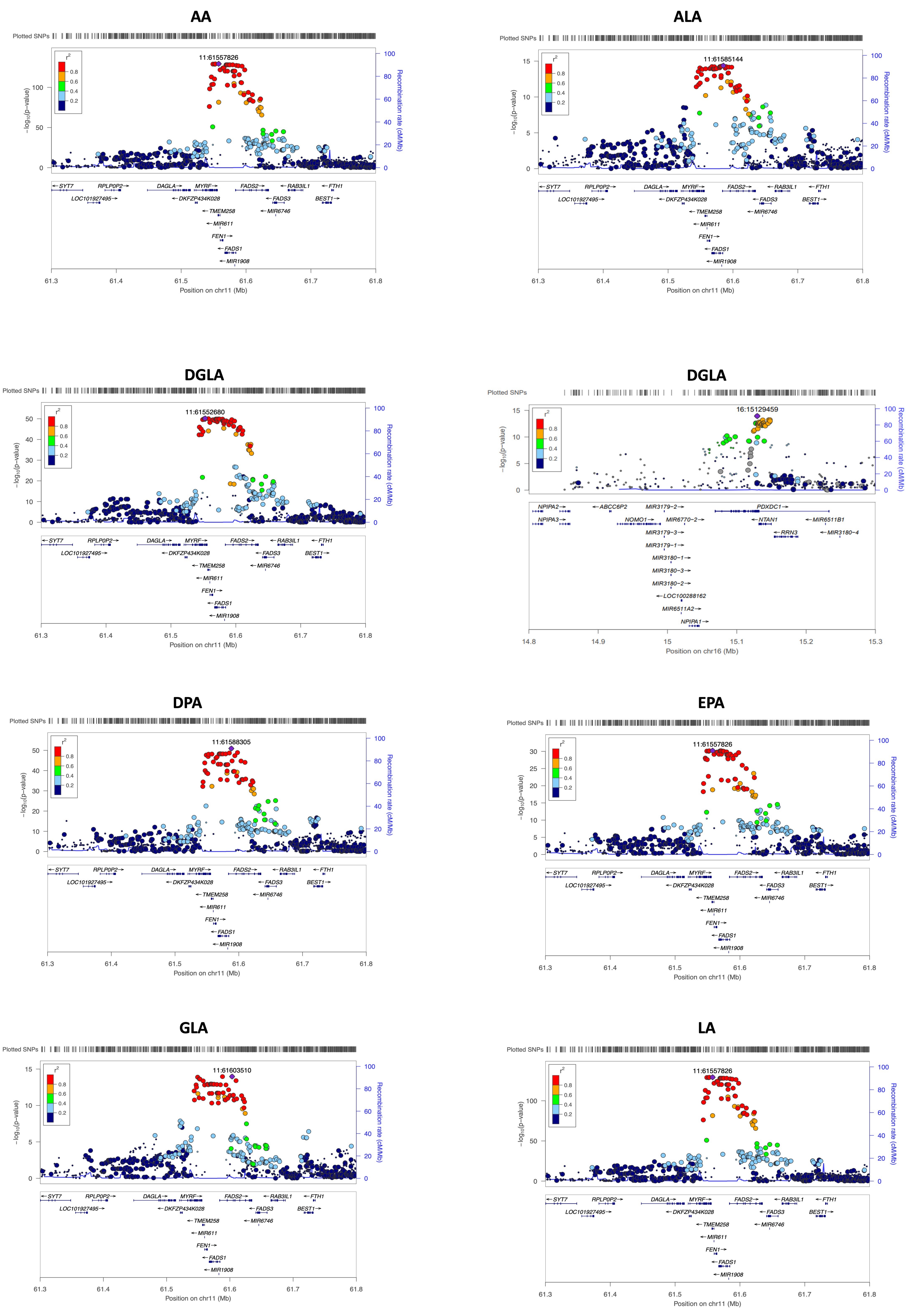
AA
Plotted SNPs
11:61557826
−log10(p−value)
Recombination rate (cM/Mb)
Position on chr11 (Mb)
ALA
11:61585144
DGLA
11:61552680
DGLA
16:15129459
Position on chr16 (Mb)
NPIPA2 ABCC6P2 MIR3179−2 PDXDC1 NPIPA3 NOMO1 MIR6770−2 NTAN1 MIR6511B1 MIR3179−3 RRN3 MIR3180−4 MIR3179−1 MIR3180−1 MIR3180−3 MIR3180−2 LOC100288162 MIR6511A2 NPIPA1
DPA
11:61588305
EPA
11:61557826
GLA
11:61603510
LA
11:61557826
SYT7 RPLP0P2 DAGLA MYRF FADS2 RAB3IL1 FTH1 LOC101927495 DKFZP434K028 FADS3 BEST1 TMEM258 MIR6746 MIR611 FEN1 FADS1 MIR1908

Supplementary Figure 3. Manhattan plot of meta-analysis of GWAS for PUFAs in the African American population.
The red line shows the genome-wide significance threshold of ($-\log_{10}(5 \times 10^{-8})$).

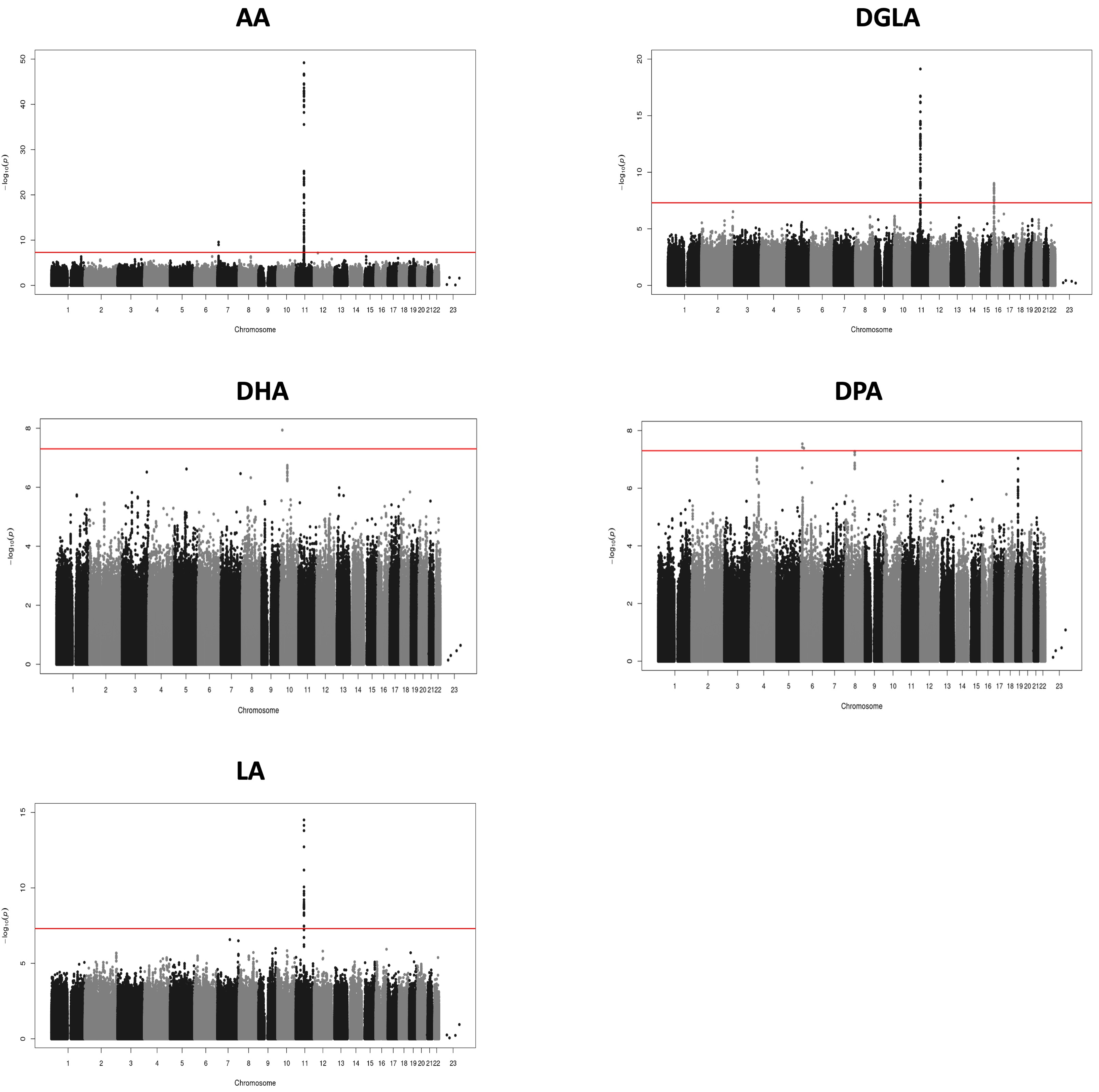
AA
DGLA
DHA
DPA
LA
Chromosome
$-\log_{10}(p)$

Supplementary Figure 4. Local association plot of most significant for each PUFAs on each chromosome in the African Americans.

Reference panel used to color LD is 1000 Genomes African (AFR) and the color scheme is red for strong linkage disequilibrium (LD; $r^2 \geq 0.8$) and blue color for lower LD.

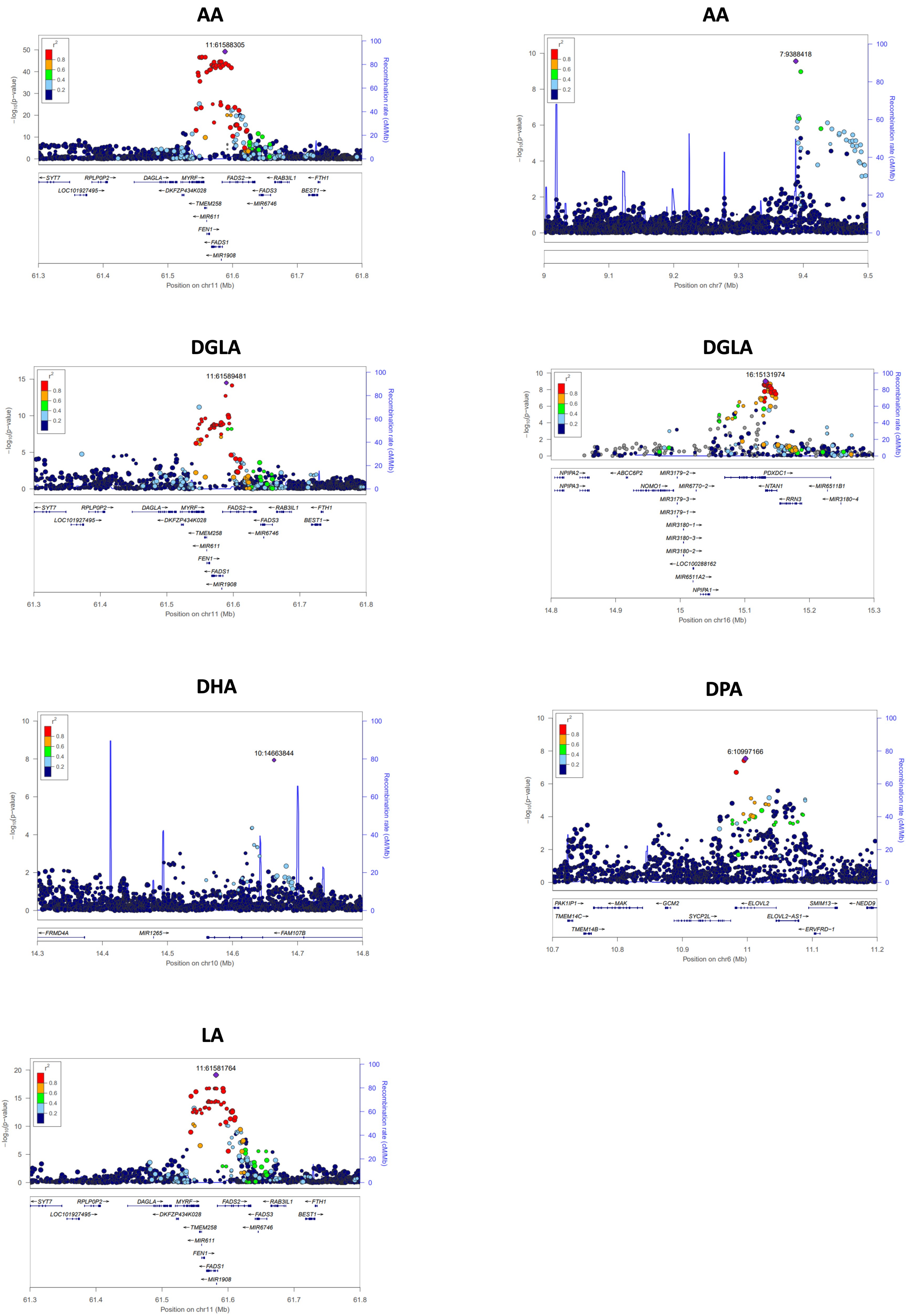
AA
11:61588305
Recombination rate (cM/Mb)
−log10(p−value)
SYT7
RPLP0P2
LOC101927495
DAGLA
MYRF
DKFZP434K028
TMEM258
MIR611
FEN1
FADS1
MIR1908
FADS2
FADS3
MIR6746
RAB3IL1
FTH1
BEST1
Position on chr11 (Mb)
AA
7:9388418
Position on chr7 (Mb)
DGLA
11:61589481
Position on chr11 (Mb)
DGLA
16:15131974
NPIPA2
NPIPA3
ABCC6P2
NOMO1
MIR3179−2
MIR6770−2
MIR3179−3
MIR3179−1
MIR3180−1
MIR3180−3
MIR3180−2
LOC100288162
MIR6511A2
NPIPA1
PDXDC1
NTAN1
RRN3
MIR6511B1
MIR3180−4
Position on chr16 (Mb)
DHA
10:14663844
FRMD4A
MIR1265
FAM107B
Position on chr10 (Mb)
DPA
6:10997166
PAK1IP1
TMEM14C
TMEM14B
MAK
GCM2
SYCP2L
ELOVL2
ELOVL2−AS1
ERVFRD−1
SMIM13
NEDD9
Position on chr6 (Mb)
LA
11:61581764
Position on chr11 (Mb)
