# Supplementary material for: Genome-wide association studies and fine-mapping identify genomic loci for n-3 and n-6 polyunsaturated fatty acids in Hispanic American and African American cohorts
Source: Commun Biol. 2023 Aug 16;6:852. doi: 10.1038/s42003-023-05219-w (PMC10432561; doi:10.1038/s42003-023-05219-w)
Supplement: Supplementary file 5 — Reporting Summary [file 42003_2023_5219_MOESM5_ESM.pdf]

nature portfolio

Corresponding author(s): Ani Manichaikul

Last updated by author(s): Jul 28, 2023

Reporting Summary

Nature Portfolio wishes to improve the reproducibility of the work that we publish. This form provides structure for consistency and transparency in reporting. For further information on Nature Portfolio policies, see our Editorial Policies and the Editorial Policy Checklist.

Statistics

For all statistical analyses, confirm that the following items are present in the figure legend, table legend, main text, or Methods section.

n/a	Confirmed	
☐	☒	The exact sample size (*n*) for each experimental group/condition, given as a discrete number and unit of measurement
☐	☒	A statement on whether measurements were taken from distinct samples or whether the same sample was measured repeatedly
☐	☒	The statistical test(s) used AND whether they are one- or two-sided<br>*Only common tests should be described solely by name; describe more complex techniques in the Methods section.*
☐	☒	A description of all covariates tested
☐	☒	A description of any assumptions or corrections, such as tests of normality and adjustment for multiple comparisons
☐	☒	A full description of the statistical parameters including central tendency (e.g. means) or other basic estimates (e.g. regression coefficient) AND variation (e.g. standard deviation) or associated estimates of uncertainty (e.g. confidence intervals)
☐	☒	For null hypothesis testing, the test statistic (e.g. *F*, *t*, *r*) with confidence intervals, effect sizes, degrees of freedom and *P* value noted<br>*Give P values as exact values whenever suitable.*
☒	☐	For Bayesian analysis, information on the choice of priors and Markov chain Monte Carlo settings
☒	☐	For hierarchical and complex designs, identification of the appropriate level for tests and full reporting of outcomes
☒	☐	Estimates of effect sizes (e.g. Cohen's *d*, Pearson's *r*), indicating how they were calculated

Our web collection on statistics for biologists contains articles on many of the points above.

Software and code

Policy information about availability of computer code

Data collection	N/A
Data analysis	Genotype imputation was carried out using the Michigan Imputation Server (https://imputationserver.sph.umich.edu) for MESA and FHS, and using IMPUTE version 2.2.2 for CHS. Statistical fine mapping of GWAS loci was conducted using SuSiE as implemented using susieR version 0.12.27. DAP-G was used to choose the starting values for SuSiE and implemented using DAP-G version 1.0.0. Bayesian colocalization analysis was implemented using R/coloc version 5.1.0.1. S-PrediXcan analysis was implemented using S-PrediXcan version 0.6.11. Gene set enrichment analysis was implemented using MSigDB v7.5.1.

For manuscripts utilizing custom algorithms or software that are central to the research but not yet described in published literature, software must be made available to editors and reviewers. We strongly encourage code deposition in a community repository (e.g. GitHub). See the Nature Portfolio guidelines for submitting code & software for further information.

Data

Policy information about availability of data

All manuscripts must include a data availability statement. This statement should provide the following information, where applicable:

- Accession codes, unique identifiers, or web links for publicly available datasets
- A description of any restrictions on data availability
- For clinical datasets or third party data, please ensure that the statement adheres to our policy

Genome-wide genotype data for the Multi-Ethnic Study of Atherosclerosis (MESA), the Framingham Heart Study (FHS) and the Cardiovascular Health Study (CHS) are available by application through dbGaP. The dbGaP accession numbers are: MESA phs000209, FHS phs000007 and CHS phs000287. Summary statistics resulting from our GWAS meta-analysis as presented in this manuscript will be available on the CHARGE Summary Results site by application through dbGaP under the accession number phs000930. Summary statistics from the prior CHARGE GWAS of n-3 and n-6 fatty acids were obtained from the CHARGE Consortium Results site. Summary statistics from the GLGC GWAS of lipid levels are available publicly at https://csg.sph.umich.edu/willer/public/glgc-lipids2021/results/trans_ancestry. All other data are available from the corresponding author (or other sources, as applicable) on reasonable request.

Human research participants

Policy information about studies involving human research participants and Sex and Gender in Research.

Reporting on sex and gender	Sex distributions are presented in Table 1 of the manuscript, and sex was included as a covariate in the primary genome-wide association study (GWAS) analyses in our manuscript.
Population characteristics	Diverse ancestry participants were included in our analyses. Hispanic Americans were included from MESA (n=1243(and FHS (n=211). African Americans were included from MESA (n=1472), CHS (n=603) and FHS (n=203). The proportion of women in these groups ranged from 50.6% in MESA/Hispanic Americans to 64.7% in CHS/African Americans. The median age ranged from 53 years in FHS/Hispanic Americans to 74 years in CHS/African Americans.
Recruitment	MESA is a longitudinal cohort study of subclinical cardiovascular disease and risk factors that predict progression to clinically overt cardiovascular disease or progression of subclinical disease.1 Between 2000 and 2002, MESA recruited 6,814 men and women 45 to 84 years of age from Forsyth County, North Carolina; New York City; Baltimore; St. Paul, Minnesota; Chicago; and Los Angeles. Participants at baseline were 38% White, 28% African American, 22% Hispanic and 12% Asian (primarily Chinese) ancestry.<br><br>CHS is a population-based cohort study of risk factors for coronary heart disease and stroke in adults ≥65 years conducted across four field centers.2 The original predominantly European ancestry cohort of 5,201 persons was recruited in 1989-1990 from random samples of the Medicare eligibility lists; subsequently, an additional predominantly African-American cohort of 687 persons was enrolled in 1992-1993 for a total sample of 5,888. Analyses were limited to those with available DNA who consented to genetic studies.<br><br>FHS is a population-based longitudinal study of families living in Framingham, Massachusetts which originated in 1948 and consisted of individuals of predominantly European ancestry.3 In 1994, the Omni Cohort 1 enrolled 507 men and women of African-American, Hispanic, Asian, Indian, Pacific Islander and Native American origins, who at the time of enrollment were residents of Framingham and the surrounding towns.
Ethics oversight	All cohort participants gave written informed consent, including consent to participate in genetic studies. All studies received approval from local ethical oversight committees including the IRBs at the University of Virginia, the University of Washington, and Dordt University.

Note that full information on the approval of the study protocol must also be provided in the manuscript.

Field-specific reporting

Please select the one below that is the best fit for your research. If you are not sure, read the appropriate sections before making your selection.

☒ Life sciences ☐ Behavioural & social sciences ☐ Ecological, evolutionary & environmental sciences

For a reference copy of the document with all sections, see nature.com/documents/nr-reporting-summary-flat.pdf

Life sciences study design

All studies must disclose on these points even when the disclosure is negative.

Sample size	The participants in this study were recruited from three population-based cohorts: the Multi-Ethnic Study of Atherosclerosis (MESA), the Cardiovascular Health Study (CHS) and the Framingham Heart Study (FHS). This manuscript focuses on HIS participants from MESA (N = 1,243) and FHS (N = 211) and AFA participants from MESA (N = 1472), CHS (N = 603) and FHS (N = 203). The sample sizes for analyses were determined based on inclusion of all participants with available genotypes for GWAS and fatty acid measures of interest.

Data exclusions	Participants who were not in the self-reported African American or Hispanic American groups of interest to this manuscript were excluded from the primary GWAS analyses.
Replication	Following statistical fine-mapping, cross-ancestry replication analyses were conducted for the most highly supported putative causal variant from each credible set using data on n-3 and n-6 PUFAs from other race/ancestry groups. The resources for replication analyses included the following:<br><br>1) European Americans (EUR): 2344 self-reported European American participants from MESA (using 1000 Genomes Phase 3 imputation, for comparison with the current study), as well as summary statistics from the previously published CHARGE GWAS meta-analysis of n-3 (n=8,866)19 and n-6 (n=8,631)20 PUFAs based on imputation from the HapMap Phase I and II,<br>2) African Americans (AFA): summary statistics from the present GWAS of PUFAs in AFA to examine cross-ancestry replication of variants identified in the present GWAS of HIS,<br><br>3) Hispanic Americans (HIS): summary statistics from the present GWAS of PUFAs in HIS to examine cross-ancestry replication of variants identified in the present GWAS of AFA, and<br><br>4) Chinese Americans (CHN): 649 self-reported Chinese American participants from MESA (using 1000 Genomes Phase 3 imputation, for comparison with the current study).
Randomization	N/A
Blinding	N/A

Reporting for specific materials, systems and methods

We require information from authors about some types of materials, experimental systems and methods used in many studies. Here, indicate whether each material, system or method listed is relevant to your study. If you are not sure if a list item applies to your research, read the appropriate section before selecting a response.

Materials & experimental systems

n/a	Involved in the study
☒	☐ Antibodies
☒	☐ Eukaryotic cell lines
☒	☐ Palaeontology and archaeology
☒	☐ Animals and other organisms
☒	☐ Clinical data
☒	☐ Dual use research of concern

Methods

n/a	Involved in the study
☒	☐ ChIP-seq
☒	☐ Flow cytometry
☒	☐ MRI-based neuroimaging
